# Considerations on immunization anxiety-related reactions in clusters

**Published:** 2014-09-30

**Authors:** Ricardo Palacios

**Affiliations:** Clinical Research and Development Manager, Division of Clinical Trial and Pharmacovigilance, Instituto Butantan São Paulo, Brazil

**Keywords:** Mass vaccination, immunization programs, drug-related side effects and adverse reactions, anxiety disorders, somatoform disorders, medical sociology, vaccines

## Abstract

A cluster of adverse events following immunization (AEFI) represents a stress test for an immunization program. The community can suspect on vaccine-related reaction leading to mistrust on the immunization program. An immunization anxiety-related reaction is one of the hypotheses to be tested and can be reasonably accepted when the vaccine-related and immunization error-related reactions are ruled out and no coincidental events can explain the cases. Immunization program approaches widely accepted to understand and respond to adverse events are root-cause analysis and systems analysis. Psychiatric cognitive frame will support the root-cause analysis assigning a causal relationship to individual temporary disorders of the affected vaccinees. Communication will focus on vaccine safety and absence of errors in the immunization program. Systems analysis addresses the whole context considering the fear spread as a systemic threat. Socio-psychological frame offers a broader opportunity to understand and respond to a specific community. Management is based on communication to change community belief in misperceptions of vaccine risks and support the idea of immunization as a causal factor, different from the vaccine. Communities can consider use of psychiatric labels, Mass Psychogenic Illness or Mass Hysteria, as an act of inconsiderateness. Labels like immunization anxiety-related reactions in clusters or collective immunization anxiety-related reactions are recommended to bridge the causal perception of the community with the result of the scientific investigation of the cases.

## Abstract (es)

Un grupo de eventos adversos después de la inmunización representa una prueba de resistencia para un programa de inmunización. La comunidad puede sospechar de reacciones relacionadas con la vacuna que llevan a perder la confianza en el programa de inmunización. Una reacción relacionada con ansiedad por la inmunización es una de las hipótesis a ser probada y puede ser aceptada en forma razonable cuando se descartan las reacciones relacionadas con la vacuna o con errores de inmunización y no hay eventos coincidentes que puedan explicar los casos. Las aproximaciones del programa de inmunización ampliamente aceptadas para entender y responder a eventos adversos son análisis de causa raíz y análisis de sistemas. El marco cognitivo psiquiátrico apoya el análisis de causa raíz asignando una relación causal a desórdenes temporales individuales de los vacunados afectados. La comunicación se enfoca en seguridad de la vacuna y ausencia de errores del programa de inmunización. El análisis de sistemas se extiende a todo el contexto considerando el temor diseminado como una amenaza sistémica. El marco socio-psicológico ofrece una oportunidad más amplia de entender y responder a una comunidad específica. El manejo está fundamentado en la comunicación para cambiar las creencias de la comunidad en percepciones equivocadas de los riesgos de la vacuna y apoyo a la idea de la inmunización como factor causa, diferenciándolo de la vacuna. Las comunidades pueden considerar las denominaciones psiquiátricas, Enfermedad Psicogénica Masiva o Histeria Masiva, como actos de desconsideración. Denominaciones como reacciones relacionadas con ansiedad por la inmunización en grupos o reacciones colectivas de ansiedad por la inmunización son recomendadas para hacer un puente entre la percepción causal de la comunidad y el resultado de la investigación científica de los casos.

"But, the myth of 'mass hysteria' can be harmful to affected persons by imposing a humiliating stigma on them as being irrational, crazy people, unlike the rest of us normal people."Bartholomew and Victor 1

## Introduction

Immunization is one of the major strategies in modern medicine for improving public health. The effectiveness of a preventive immunization program is the result of the vaccine's biological activity on each individual, but a program's effectiveness may also be due to the protection extended to the whole community through herd immunity. Thus, a person's decision to get vaccinated or not is more than an individual choice; it may compromise a vaccination campaign and therefore impact a community's public health. Furthermore, if a vaccine's reputation is affected, the whole immunization program may be jeopardized. 

The target population of an immunization program is usually composed of healthy individuals, who accept a mild discomfort in order to avoid the risk of getting a disease. The way in which individuals or communities perceive discomfort or risk can affect their willingness to be vaccinated. 

A cluster of adverse events following immunization (AEFI) represents a stress test for an immunization program. Procedures for vaccine-related reactions and immunization error-related reactions are usually in place in all vaccination sites. This article aims to make considerations about one of the most difficult challenges for an immunization program: immunization anxiety-related reactions occurring in clusters.

### How can one prove that a cluster of AEFIs may be due to immunization anxiety-related reactions?

Simple answer; it is very unlikely that anybody can prove this. So what can be done? According to the Council for International Organizations of Medical Sciences (CIOMS) and the World Health Organization (WHO), there are two kinds of reactions that can occur as an AEFI: those related to the vaccine (vaccine-related reactions), and those related to immunization procedures (immunization- related reactions). The latter AEFI is the coincidence of events not related to either the vaccine or with the immunization. This classification is presented in Table 1 2 ^,^ 3. Vaccine-related reactions are attributable either to the vaccine as a biopharmaceutical product due to unexpected or expected adverse reactions to the product or associated to quality defects in the manufacturing process. Immunization-related reactions do not have a casual relationship with the vaccine, as a product; indeed, they are temporally associated with the immunization procedures. They can occur due to an immunization error or because of anxiety associated to the immunization process itself. 

**Table 1.**
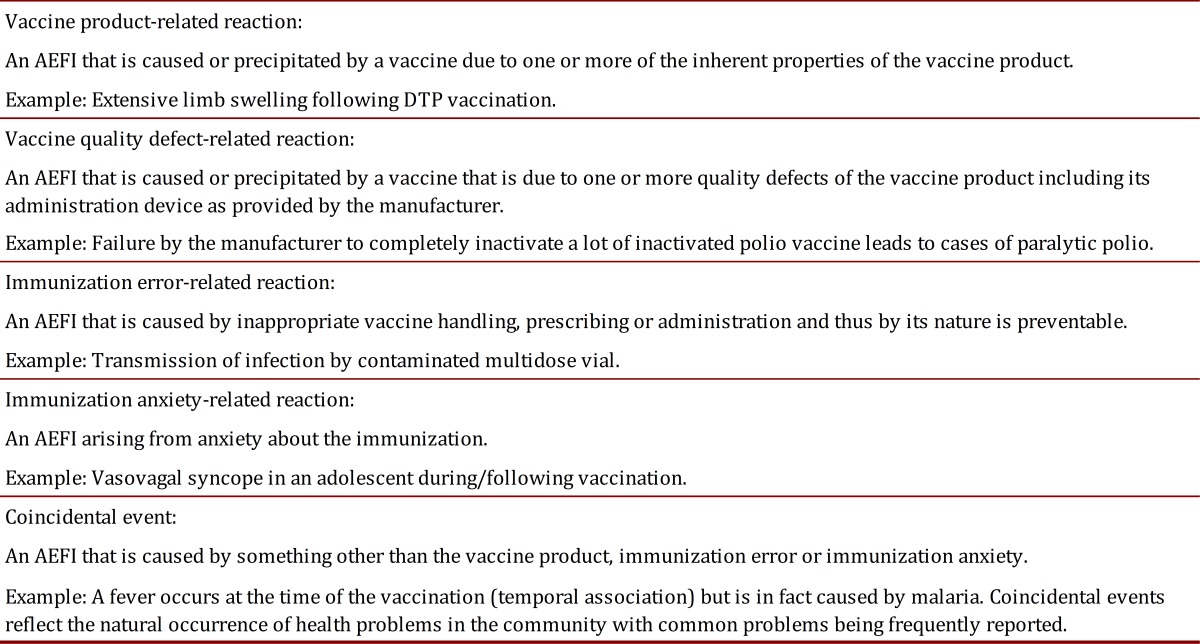
Classification of the adverse events following immunization (AEFI) according to the Council for International Organizations of Medical Sciences and the World Health Organization (Reproduced form the WHO Vaccine Safety Basic e-Learning Course^3)^

Vaccine quality, or defect-related, reactions are the easiest to be clarified. If the product has a technical specification and the samples of the product associated with the cluster reactions have a deviation from the technical specification, such evidence can build a strong case against the vaccine's quality. Other cases exposed to the same quality deviation lot can confirm the causality.

When an immunization error occurs, an audit can provide support about potential failures that originated from immunization error-related reactions, i.e. cold chain failures.

The pre-licensure clinical research detects most of the vaccine product-related reactions, which are in the package insert as expected adverse reactions. Although vaccines are classified as one of the safest medical interventions, they may be eventually associated with unexpected adverse reactions, which will only be observed after their widespread use in the general population and/or in populations with specific genetic background. Nevertheless, such reactions are usually grouped for further investigation and do not appear in clusters

Coincidental events can also trigger an AEFI in groups of vaccinees and should also be considered. Background health surveillance can provide clues to follow on the investigation of the AEFIs. 

Finally, there are immunization anxiety-related reactions. Unfortunately, no diagnostic test can assure that anxiety associated to immunization is the cause of a reaction. This fact increases the possibility of conflict between affected communities and immunization programs 4.

There are three basic attributes of causality in epidemiology: association, time order and direction 5. For affected communities, it is easy to find those attributes in episodes of immunization anxiety-related reactions because the event occurs just after the person receives the vaccine. The vaccine becomes the "usual suspect," since the community already has some level of mistrust. The immunization program is challenged to provide evidence of what happened 6.

According to Popper, the falsification, or disproving, of hypotheses is the methodological basis of scientific knowledge in "hard sciences." Instead of looking for evidence to verify a hypothesis in the commonsense approach, hypothetico-deductive method advocates for designs that aim to refute a hypothesis. Thus, a null hypothesis should be rejected so that an alternative hypothesis can be accepted. When an alternative hypothesis for a cluster of AEFIs is formulated, other options should be rejected; this way the alternative hypothesis is strengthened because it successfully resists several rounds of falsification 7. In fact, the hypothesis of immunization anxiety-related reaction is not proven, but becomes stronger when the other, alternative explanations are rejected. 

Two types of evidence can support a claim of causality of an AEFI: mechanistic and epidemiological. Mechanistic evidence includes clinical or biological mechanisms that explain an event. Observational and interventional studies provide epidemiological evidence 8. Regarding mechanistic evidence, there are reports of collective anxiety in several settings with different vaccine products after immunization 6 ^,^ 9 ^-^ 14. However, the planning of epidemiological studies is not feasible due to the unexpected nature of the phenomenon. Nevertheless, the mechanistic evidence is strong enough to provide proof that immunization anxiety can cause reactions in clusters. 

In analogous situations, some features usually appear as part of what medical literature calls "mass psychogenic illness" or "mass hysteria". The reports include a large variety of trigger stimuli such Coca-Cola 15, odors or fear of being exposed to a noxious agent without evidence of such exposure 1 ^,^ 16 ^,^ 17. Such cases Page *et al*.^16^, have validated criteria to determine whether a mass psychogenic illness occurred after exposure to a potentially toxic chemical agent (Table 2). Of note, incidents occurring in schools and healthcare facilities are more likely to be classified as mass psychogenic illnesses. These criteria might also guide the assessment of AEFI, but validation processes did not include such incidents, and therefore can provide limited guidance in immunization anxiety-related reactions. A recently published quasi-experiment reported how experienced blood donors were more likely to have a vasovagal reaction (OR 2.5 IC_95_ 1.16-5.39) if they watched another donor being treated for vasovagal symptoms 18. 

**Table 2.**
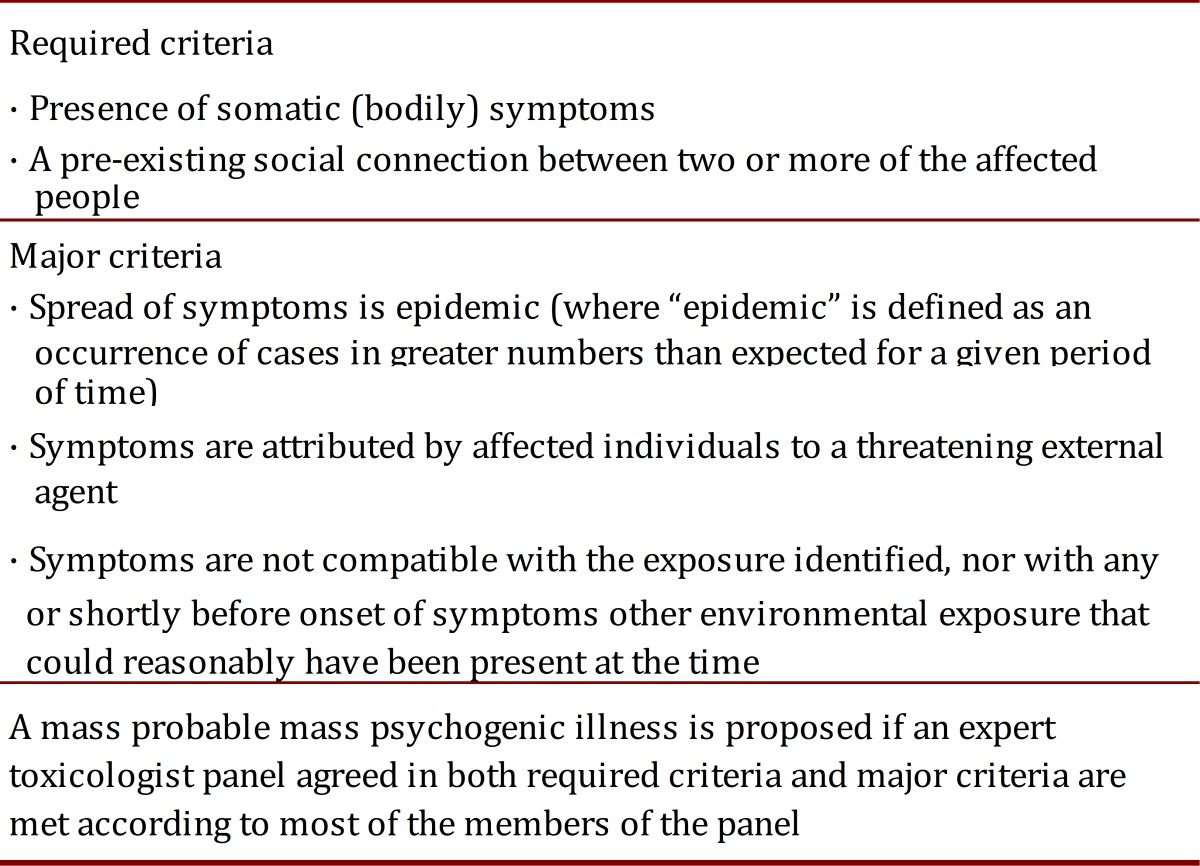
Criteria to define Mass Psychogenic Illness after a potential chemical exposure incident (Modified from Page *et al* ^6)^.

Once a cluster of immunization anxiety-related reactions arises as an alternative hypothesis, investigators' efforts should aim to reject it 7. The null hypothesis is one raised by affected people/communities: that the vaccine is associated to the cluster of adverse events. Thus, vaccine-related events are the first to be refuted. This approach is also valuable in terms of risk management: if the null hypothesis is not rejected, urgent action can prevent additional adverse events from happening to a population potentially exposed to the same vaccine or lot of vaccine. Rejection of immunization errors will rule out a hypothesis that might confound with the null and the alternative hypotheses. Once the abovementioned hypotheses and coincidental phenomena are refuted, the alternative hypothesis of immunization anxiety-related reactions can be reasonably accepted.

### How to handle a cluster of AEFIs due to immunization anxiety-related reactions

There are two approaches widely accepted to understand and respond to adverse events: root-cause analysis and systems analysis 19. In a root-cause analysis, the investigation tracks the event until the problem is discovered in order to control it and avoid new events. A systems analysis aims to assess whether the system is reaching its purposes, or not, and what the gaps are between reality and expectations. These approaches can have radical consequences on AEFIs in clusters handling.

For a root-cause analysis, it is important to understand everything from the cause to the ultimate consequences. In this way, psychiatry provides a cognitive frame for explanations on the cause of AEFI not related to vaccines or due to immunization errors. Mass hysteria or mass psychogenic illnesses are the labels for these kinds of events, assigning a causal relationship to individual temporary disorders of the affected vaccinees, out of the immunization program's control. In the same sense, communication to the public focuses on the reinforcement of controlling the factors affecting related processes, such as safe vaccines and no errors in the immunization program.

Systems analysis obliges one to address the whole context of the AEFIs and not just individual causes. A cluster of AEFIs is a systemic threat to the purpose of the immunization program because it might spread fear and decrease overall immunization rates. In this sense, a socio-psychological frame offers a broader opportunity to address why the phenomenon occurred in a specific community and how to manage it. The labels of immunization anxiety-related reactions in clusters or of collective immunization anxiety-related reactions reflect this cognitive frame; the situation does not occur as an unfortunate junction of abnormal personalities but as a consequence of shared beliefs and representations of immunization, which contributes in produces anxiety in a community. The name of the phenomenon also acknowledges that the immunization process triggers the problem and supports the idea of immunization as a causal factor, different from the vaccine. Communication should be the basis of the management of the phenomenon by promoting changes in beliefs of the community, besides providing support to the affected people and groups. A comparison between root-cause and system analyses in terms of understanding and managing clusters of immunization anxiety-related reactions is detailed in Table 3, adapted from the theory proposed by Bartholomew and Victor 1. In fact, immunization program and health authorities' responses are actually a mix of both approaches and the predominance of one or another response can influence the outcome. 

**Table 3.**
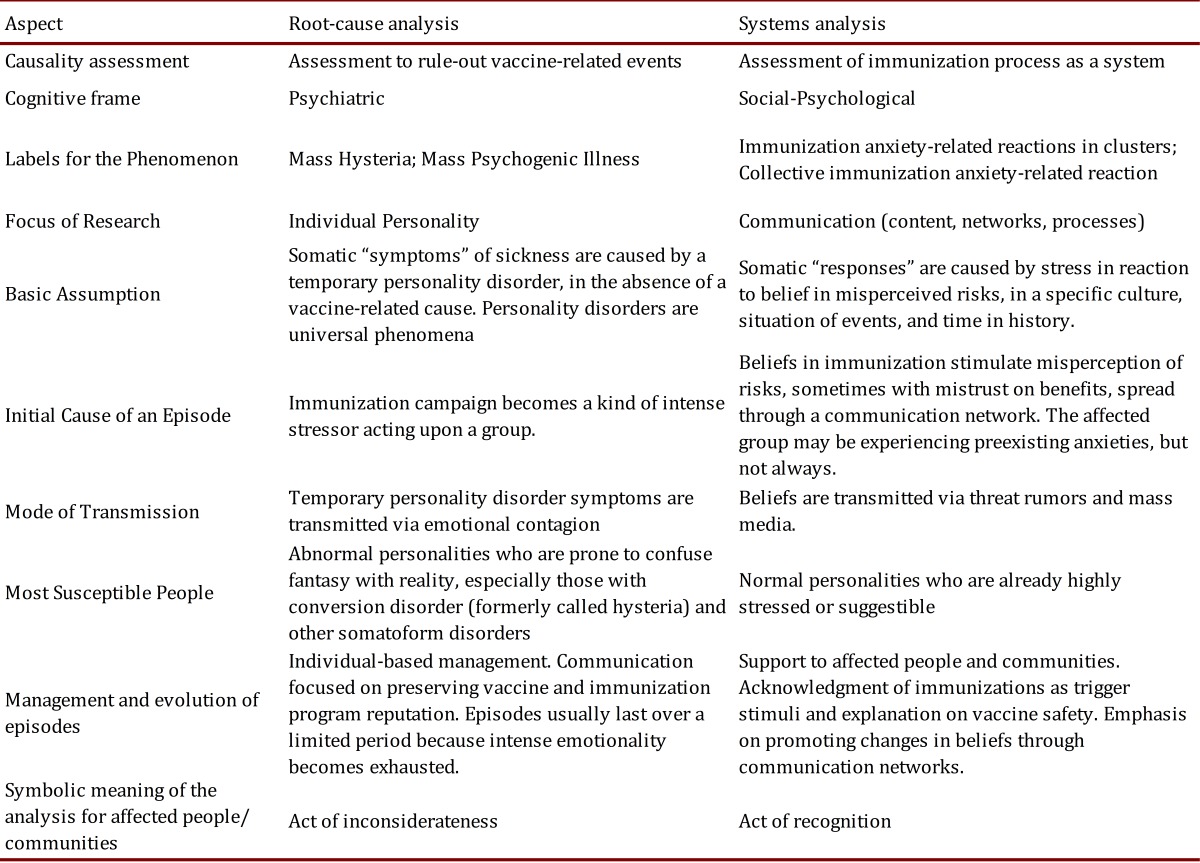
Comparison of root-cause and systems analyses for investigation of a case series of immunization anxiety-related reactions in a community (Modified from Bartholomew and Victor ^1)^.

A cluster of unexpected immunization anxiety-related reactions is usually conflictive. Vaccines, relatives and communities can feel deceived because they believe they have received harm instead of benefit from the immunization process. If the causal association is focused on intrinsic personality features of the affected vaccines, this confrontation tends to arise because the community does not have a satisfactory alternative hypothesis or one better than the current association with the vaccine; this mistrust reinforces misperceptions. Using the wording "psychogenic illness" or "hysteria" makes a collective representation of "symptoms" created in the minds of the patients 1 ^,^ 20. In terms of the symbolic dimension of a conflict 21, this is an act of inconsiderateness, equivalent to a moral insult. Therefore, those terms should be avoided and replaced by the standard AEFI typology proposed by CIOMS-WHO: immunization anxiety-related reactions, with the terms "collective" or "in clusters" to indicate the actual presentation. This terminology can easily bridge the causal perception of the community showing the results of the scientific investigation of the cluster of AEFIs. Moreover, patients are considered normal individuals having anxiety reactions to an actual stimulus within a specific context. By the end, the symbolic meaning of this approach is an act of recognition that will lead to the resolution of the conflict 21. This recognition, contrary to inconsiderateness, is aligned with public service, one of the key values of an immunization program.
